# A synthetic approach to the Holiday Climate Index for the Mediterranean Coast of Türkiye

**DOI:** 10.1007/s00484-024-02704-7

**Published:** 2024-06-05

**Authors:** Başak Bilgin, Sevil Acar, Zekican Demiralay, Nazan An, M. Tufan Turp, M. Levent Kurnaz

**Affiliations:** 1 Department of Tourism Administration, Boğaziçi University, 34342 Istanbul, Türkiye; 2 Center for Climate Change and Policy Studies, Boğaziçi University, 34342 Istanbul, Türkiye; 3 Computational Science and Engineering, Boğaziçi University, 34342 Istanbul, Türkiye; 4 Meteorological Institute, Ludwig-Maximilians-University, 37 80333, Munich, Germany; 5 Department of Physics, Boğaziçi University, 34342 Istanbul, Türkiye

**Keywords:** Holiday Climate Index, Coastal tourism, Tourism climate indices, Climate change, Panel data analysis

## Abstract

Climate change affects the comfort level of tourists visiting coastal areas. Researching these impacts is important for a more comprehensive understanding of climate change and for developing effective adaptation solutions. Considering this fact, the study derived the Holiday Climate Index (HCI: Coast, HCI: Urban, and HCI: Combined) in the Mediterranean coastal provinces of Türkiye from 1976 to 2020. Utilizing the derived indices, the effects of climate-related holiday comfort on the number of tourist arrivals as well as on overnight stays between 1976 and 2020 were examined by panel data analysis. The study examined how comfort patterns could change during the period 2026–2050 under the pessimistic RCP8.5 scenario. It was detected that the comfort level significantly and positively affects the number of arrivals and overnight stays of tourists. Besides, comfort levels were found to deteriorate in the period 2026–2050 compared to the reference period, 1976–2020. As the comfort conditions get worse, the number of tourist arrivals and overnight stays is expected to decline in the future. It is envisaged that the results of the study can be useful for tourists, tourism professionals, operators, other stakeholders, and policymakers.

**Supplementary Information:**

The online version contains supplementary material available at 10.1007/s00484-024-02704-7.

## Introduction

Climate change is a long-term change in the world’s climate system caused by natural and anthropogenic factors. It is a phenomenon caused mostly by industrial activities, causing substantial changes in temperature, precipitation, air movements, and sea level (IPCC 2021). The Paris Agreement aims to limit global warming to 1.5 °C but achieving this goal has become increasingly difficult (IPCC 2023). The effects of warming may persist for years or centuries (Nicholls et al. 2018). It has significant impacts on society and various sectors of the economy, including tourism, in the form of decreased number of tourist arrivals, deterioration of settlements and infrastructure, increased mortality rates, water and food insecurity, poor welfare, and so on (IPCC 2022). Climate change affects human health through increased heat stress, respiratory issues, and infectious diseases (IPCC 2022; WHO 2016). It significantly affects human comfort levels, with rising temperatures, humidity, air pollution, and the frequency and intensity of extreme weather events causing discomfort. Sustainable development aims to mitigate the devastating effects of climate change and enable us to adapt to these effects. The consequences of climate change constitute an obstacle to development, and sustainable development is of critical importance in solving this problem. Tourism, by its nature, is one of the sectors that develop in harmony with nature, feed on nature and is most affected by even small or instantaneous changes in nature. A more macro impact, such as climate change, which completely changes life on earth, is expected to bring about a significant change in the structure, types, and regions of tourism. Although adapting to the current situation and future scenarios is considered an option, failure to achieve sustainability goals may restrict these adaptation practices as temporary and costly options.

Tourism is a critical and rapidly expanding economic sector with a continuously increasing impact on the global economy. The COVID-19 pandemic in 2020 resulted in a dramatic 58% decrease in international tourist arrivals compared to the previous year. Despite this decline, the tourism sector remains a major contributor to the global economy, representing 10% of global GDP and accounting for one-tenth of all jobs worldwide (UNWTO 2021). The travel and tourism industry made a sectoral contribution of $9.2 trillion (10.4% of global GDP) and provided 334 million jobs (10.6% of global employment) in 2019 (WTTC 2021).

The weather has the potential to facilitate or impede tourism activities (Gómez-Martín, 2005). Extreme weather events can lead to transportation delays, cancellations, and accidents, posing risks to tourist safety. Climate change could exacerbate these risks in the future. Tourism is vulnerable to climate change due to its close relationship with the environment and climate. One instance of the climate’s impact on tourism and the resources accessible to individuals is evident in factors such as weather perception, safety, and thermal comfort (Scott et al. 2012). Tourism faces challenges from climate change, making it crucial to assess weather variability effects, design effective policies, and evaluate potential economic impacts. The relationship between tourism and environmental changes is bi-directional, with environmental changes impacting tourism and tourism activities also contributing to environmental changes. Global tourism, a trillion-dollar industry, significantly affects the environment through infrastructure, local resource demands, and habitat destruction. Also, the tourism industry contributes to environmental change through carbon emissions, especially because of tourism-related mobility (Lenzen et al. 2018).

Over 400 million international tourists visited the Mediterranean Basin in 2019 (UNWTO 2022), causing considerable pressure on coastal ecosystems and local populations. The region is a popular tourist destination with exceptional sociocultural and environmental diversity, transformed over thousands of years due to human activities. It receives one-third of international tourists, and France, Spain, Italy, Türkiye, and Greece are the most popular tourist destinations (UNWTO 2021). Climate change threatens all Mediterranean countries in socio-economic sectors since the tourism industry in the Mediterranean Basin, which is predominantly located in coastal areas, is highly vulnerable to the impacts of climate change. The basin is expected to experience warming that surpasses global rates by over 20%, and summer warming is projected to be 40% faster than the global average (Lionello and Scarascia 2018). In addition, climate models consistently project less precipitation for the basin (IPCC 2022). The Mediterranean Basin countries will experience a decrease in tourism due to increasing temperature extremes and the occurrence of extreme climate events (IPCC 2022; Kutiel 2019; Dos Santos et al. 2020). It is crucial to identify these effects to facilitate adaptation and mitigation efforts because climate change has a significant impact on visitors by exacerbating thermal discomfort (IPCC 2021; Zinzi and Carnielo 2017) requiring reliable information for tourists and organizers.

Various efforts have been undertaken to determine the ideal climate conditions for tourism in general and specific tourism activities. An index approach is beneficial for understanding the relationship between climate variables and tourism in relation to climate due to its complexity and multifaceted nature. During the 1960s and 1970s, there was a noticeable expansion in the global tourism sector. Following this development, various studies have started to investigate the correlation between destination climate and tourism demand (Perry 1972; Mieczkowski 1985; de Freitas 1990; Smith 1990). Several attempts have been made to measure the climatic comfort experienced by tourists from a biometeorological standpoint. This involves assessing weather parameters and the level of outdoor thermal comfort. Mieczkowski (1985) identified the necessity for an index to assess the climatic conditions of tourist destinations. He developed the Tourism Climate Index (TCI), which is the first to assess the correlation between tourism and climate and favourable and unfavourable climate conditions. Numerous tourism climate indices were developed following the creation of TCI, such as the Beach Climate Index (BCI) (Morgan et al. 2000); the Climate Index for Tourism (CIT) (de Freitas et al. 2008); the Modified Tourism Climate Index (MCIT) (Yu et al. 2009); the Relative Climate Index (RCI) (Li et al. 2018). HCI was developed as a response to the limitations of these indices (Tang 2013; Scott et al. 2016; Rutty et al. 2020). It is based on objective criteria rather than subjective opinions. The determination of rating scales and weights for sub-indices is informed by existing literature on visitors’ climate preferences (Tang 2013; Scott et al. 2016; Rutty et al. 2020; Demiroglu et al. 2020). Several studies have shown that HCI addresses the limitations of TCI and aligns better with visiting patterns in urban and beach destinations (Tang 2013; Scott et al. 2016; Rutty et al. 2020; Demiroglu et al. 2020; Yu et al. 2021; Samarasinghe et al. 2023).

This study aimed to assess the top ten Mediterranean coastal provinces in Türkiye that are most popular among tourists during the summer season (Adana, Antalya, Aydın, Balıkesir, Çanakkale, Edirne, Hatay, İzmir, Mersin, Muğla). The primary objective was to analyse the evolution of human comfort patterns in these provinces from 1976 to 2020. The second objective of this study was to examine the impact of climatic characteristics on tourism activities in the listed ten provinces. The third objective was to examine the future trends in HCI indices for the study area. The study used three derived HCI indices (HCI: Urban, HCI: Coast, and HCI: Combined) to analyse the impact of climate change on tourism patterns. Then, these indices were regressed on the number of tourist arrivals and overnight stays using panel data analysis. Finally, future (2026–2050) trends in HCI indices for ten provinces were analysed. 1976–2020 was determined as the reference period in the study. All available data was used for the section where the relationship between the number of tourist arrivals and overnight stays and tourist comfort has been examined by regression analysis, that is, 1976–2020.

To the best of our knowledge, this study is the first one that examines the relationship between the HCI and the number of tourist arrivals and the days of overnight stays on the Mediterranean coasts of Türkiye. The analysis of the impacts of climate change on the comfort levels of tourists is beneficial in order to gain a thorough comprehension of the potential effects of climate change on the tourism industry and to formulate efficient strategies. The study intends to fill the gap in the regional analyses of climate change regarding its implications for holiday comfort and tourism activity. The organization of the paper is as follows: Sect. 2 introduces the data and the methodology. Section "Data" introduces the results regarding HCI trend analysis of the number of arrivals and overnight stays between 1976–2020 and panel data analysis, and the future trends of HCI-derived indices between 2026–2050. Section "Domain" discusses the implications of the study.

## Materials and methods

### Data

#### Domain

**Fig. 1:**
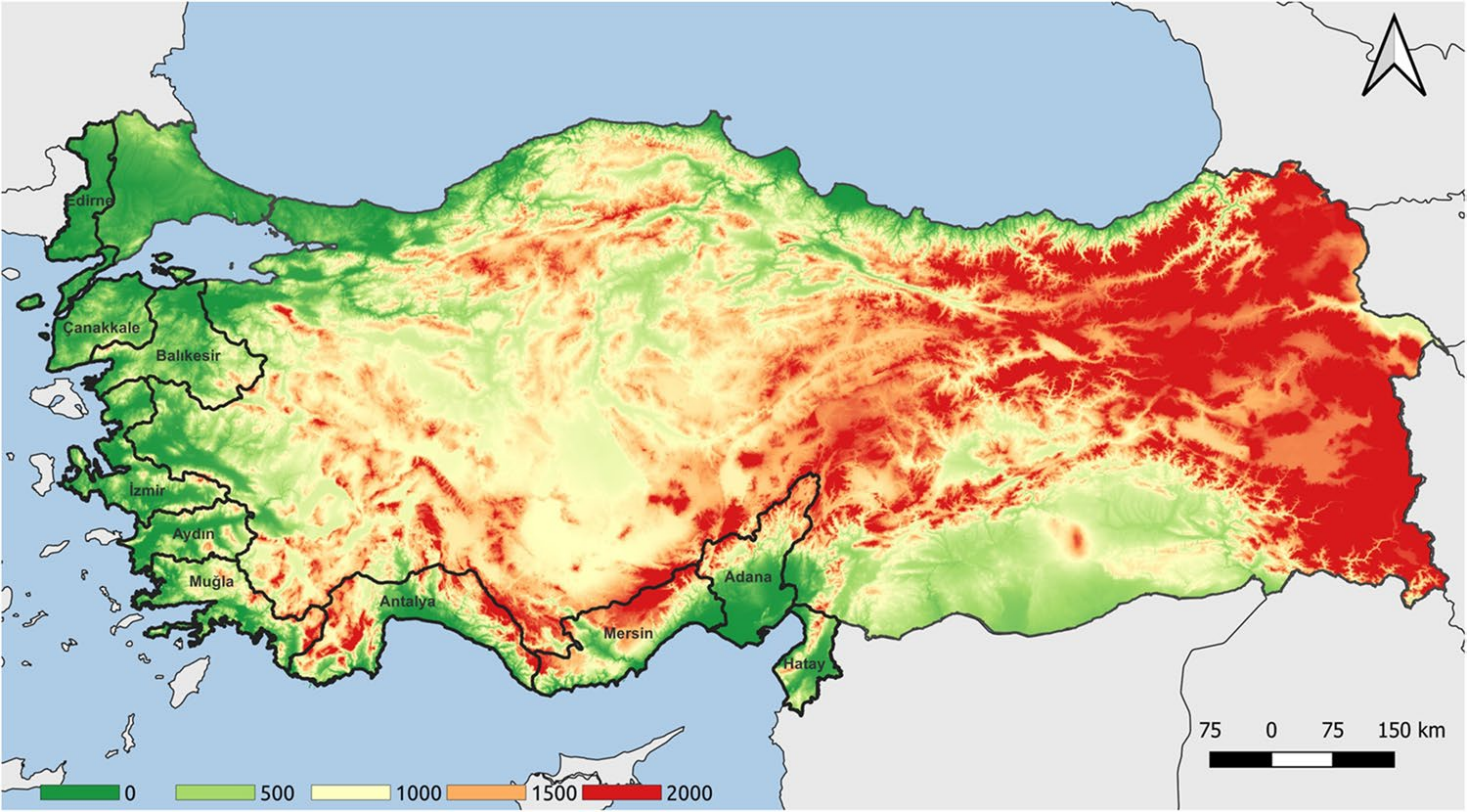
The elevation map showing the study area

According to the Köppen-Geiger classification, the provinces in this study are in the temperate (C) climate class. In this group, generally humid climatic features are seen. The provinces examined in the study (Adana, Antalya, Aydın, Balıkesir, Çanakkale, Edirne, Hatay, İzmir, Mersin, Muğla) are located in the south-southwest and west of Türkiye (Fig. 1) and have a Mediterranean climate with arid and very hot summers (Türkeş 2022). Mediterranean and Aegean coasts have warm winters, with high temperatures during the day (above 25 °C). Swimming, sand or sea bathing, and water sports are available for 200–250 days on the Mediterranean and South Aegean coasts and 150–180 days on the North Aegean coasts (Doğanay and Zaman 2021).

#### Climate data

The Max Planck Institute Earth System Model Mixed Resolution (MPI-ESM-MR) and the Met Office Hadley Center Earth System Model (HadGEM2-ES) were used as Global Circulation Models (GCMs) in this study. Comparative results were obtained with these two models. In order to obtain regional results, GCM data were dynamically downscaled to 10 km grid resolution using the regional climate model RegCM4.4 (Giorgi et al. 2012) developed by the Abdus Salam International Centre for Theoretical Physics. High-resolution climate data was obtained from the RegCM4.4 regional climate model by using low-resolution MPI-ESM-MR (Giorgetta et al. 2013) and HadGEM2-ES (Collins et al. 2008; Jones et al. 2011) global climate model outputs as input. It is widely used in climate-related research around the world (Turp et al. 2014; Sylla et al. 2016; Ozturk et al. 2015, 2017, 2018; An et al. 2020). Projection studies were conducted using RCP8.5, a scenario in which greenhouse gas emissions continue to increase and global CO_2_ concentration will reach approximately 936 ppm by 2100 (van Vuuren et al. 2011; Riahi et al. 2007, 2011).

### Methodology

#### HCI computation

The Holiday Climate Index (HCI) was developed in 2013 (Tang 2013) to improve upon existing tourism climate indices, specifically the TCI, by addressing their limitations, and it was improved later (Scott et al. 2016). HCI uses the existing literature on tourists’ climate preferences from recent surveys to determine the rating scales and weights of sub-items. There are two distinct categories of HCI, specifically HCI: Urban and HCI: Coast. These indices incorporate different weights for thermal comfort and cloud cover in order to be customized to the specific climatic requirements of different tourism segments. HCI: Urban was created for urban tourism (Tang 2013; Scott et al. 2016). HCI: Coast was created based on research regarding the climate preferences of coastal visitors (Rutty et al. 2020). In this study, while calculating HCI, the HCI: Coast formula was applied to the grid points corresponding to the coastal districts of each province, the HCI: Urban formula for non-coastal districts. Then, HCI: Combined was determined by computing the average of all grid points within the borders of the provinces to obtain the provincial average. The decision to calculate HCI: Combined was made to ensure robust results, as considering an entire province solely as an urban or coastal region would be insufficient.

HCI uses four climate variables related to three tourism-related factors: relative humidity (%) for thermal comfort (TC), cloud cover (%) for aesthetics (A), precipitation (mm) and wind (km/h) for physical (P) components. Each climate variable is graded on a scale of 0 to 10, and the HCI index score ranges from 0 to 100. The HCI: Coast, HCI: Urban, and HCI: Combined are calculated as follows:

1 $$HCI: Coast = 2(TC) + 4(A) + 3(P) + (W)$$

2 $$HCI: Urban = 4(TC) + 2(A) + 3(P) + (W)$$

3 $$HCI: Combined = HCI: Coast x c1 + HCI: Urban x c2$$

#### Panel fixed effects model

The objective of this section was to investigate the impact of climate-related comfort on tourism activities in the Mediterranean provinces of Türkiye, specifically focusing on the aspect of human comfort. The study employed panel data analysis using a fixed effects specification to examine the relationship between the number of tourist arrivals and days of overnight stay, and the coastal, urban, and combined HCI indices derived for the Mediterranean coastal provinces of Türkiye. Panel data refers to a collection of data that allows for the observation of the behaviour of entities (*i*) over a specific period of time (*t*). When the observation units from a cross-sectional sample are inspected many times, the resulting observations form a panel or longitudinal data set (Dougherty 2016). The utilization of this approach enables the management of individual heterogeneity by facilitating the analysis of variation and reducing the presence of linear correlation among variables (Gujarati 2022).

The utilization of panel data in this research facilitated the investigation of the impact of holiday climate indices on tourism activities at the province level and throughout a broad temporal range (1976–2020). The provinces with a coastline to the Mediterranean, covering the Aegean Sea in Türkiye’s Mediterranean areas that are considered to have the highest number of tourists (Ministry of Culture and Tourism, n.d.) were chosen by a pre-determined threshold. It was set to include the top 10 coastal provinces with the greatest influx of tourists in these regions.

The proposed model can be stated in the following form:

4 $${y}_{it}=a+{X}_{it}\beta+{u}_{it}$$

In the given equation, for *i* = *1,…, N* and *t* = *1,…, T,* where *y*_*it*_ is the dependent variable, *X*_*it*_ is the vector of independent variables, *α* is the constant term, *β* is the coefficient of HCI, and *u*_*it*_ is the error term, formatted as follows:

5 $${u}_{it}={\mu}_{i}+{\nu }_{it}$$

where *μ*_*i*_ is the unobservable individual-specific effects (i.e., fixed effects), and *ν*_*it*_ is the remainder disturbance. *μ*_*i*_ is time-invariant, and it incorporates any individual-specific effects that are omitted from the regression. *ν*_*it*_ varies from individual to individual and over time and can be interpreted as the usual disturbance in the regression. The fixed effects *μ*_*i*_ cannot be directly predicted. Each *μ*_*i*_ is a discrete constant linked with a specific group or cross-section. Thus, the constant term for every cross-section is α + *μ*_*i*_. The remaining disturbances *ν*_*it*_ exhibit stochastic behaviour, are independent from each other, and follow a uniform distribution (*iid*). The explanatory variables are independent of *ν*_*it*_. The fixed effects model is the recommended choice when all independent variables are conditionally associated with individual effects, while the random effects model is applicable when all independent variables are exogenous to random individual effects (Mundlak 1978). In the current analysis, we relied on Hausman (1978) test based on the difference between the fixed and random effects estimators, and consequently fixed effects estimator was preferred.

The analysis was conducted taking the number of tourist arrivals and the days of overnight stays as the dependent variables for the ten provinces from 1976 to 2020. A 6-month and annual analysis was conducted on the HCI: Coast, HCI: Urban, and HCI: Combined. The sea water temperature in the Mediterranean coasts during May–October can reach 20–28 °C, contributing to their extended holiday season (Zengin 2006). Therefore, the study examined the six months with the highest number of tourist visits, which are considered the summer tourism season for the Mediterranean coasts in Türkiye (Gao and Giorgi 2008; Türkeş et al. 2009; Rutty and Scott 2010; Cook et al. 2016; Demiroglu et al. 2017). The dynamically downscaled MPI-ESM-MR and HadGEM2-ES models were used to analyse these months, along with the annual data.

## Results

### HCI trend analysis for the 1976–2020 period

In this section, a trend analysis was performed to evaluate the changes in the HCI indices between 1976 and 2020. Trend analyses were examined on annual and 6-month basis in both models (MPI-Annual, HG-Annual, MPI-6-Month, and HG-6-Month). The Mann–Kendall test and Sen’s slope estimator are important for trend analysis. They are widely used for detecting and quantifying trends in various environmental time series data, such as temperature, rainfall, runoff, and groundwater levels, which is crucial for environmental monitoring and resource management (Partal and Kahya 2006; Atta-ur-Rahman and Dawood 2017; Rahman et al. 2017; Güçlü, 2018; Perera and Rathnayake, 2019). In conducting the trend analysis for the Holiday Climate Index (HCI) in our study, we examined time series data from popular coastal provinces in Türkiye. We did not employ the Mann–Kendall test or Sen’s slope estimator for trend analysis because our approach focused on comparing trends observed in different regions rather than applying specific statistical tests. With this method, we distinguished patterns and differences in HCI trends among the provinces examined in the study. Table 1

**Table 1:** The ratings of thermal comfort (based on humidex), aesthetic (based on cloud cover), precipitation, wind, and HCI: Coast scores

Thermal Comfort (TC)		Aesthetic (A)		Precipitation		Wind		HCI: Coast Score	
**Humidex**		**Cloud Cover**							
	**Rate**	**%**	**Rate**	**mm**	**Rate**	**km/h**	**Rate**		**Rate**
(-∞, 10) [10, 15) [15, 17) [17, 18) [18, 19) [19, 20) [20, 21) [21, 22) [22, 23) [23, 26) [26, 28) [28, 31) [31, 33) [33, 34) [34, 35) [35, 36) [36, 37) [37, 38) [38, 39) [39, ∞)	-10 -5 0 1 2 3 4 5 6 7 9 10 9 8 7 6 5 4 2 0	[0, 1) [1, 15) [15, 26) [26, 36) [36, 46) [46, 56) [56, 66) [66, 76) [76, 86) [86, 96) [96, 100]	8 9 10 9 8 7 6 5 4 3 2	[0, 0.01) [0.01, 3) [3, 6) [6, 9) [9, 12) [12, 25) [25, ∞)	10 9 8 6 4 0 -1	[0, 0.6) [0.6, 10) [10, 20) [20, 30) [30, 40) [40, 50) [50, 70) [70, ∞)	8 10 9 8 6 3 0 -10	[0, 20) [20, 40) [40, 50) [50, 60) [60, 70) [70, 80) [80, 90) [90, 100)	Dangerous Unacceptable Marginal Acceptable Good Very Good Excellent Ideal

^*^ adapted from Tang (2013); Scott et al. (2016); Rutty et al. (2020);Demiroglu et al. (2020)

**Fig. 2:**
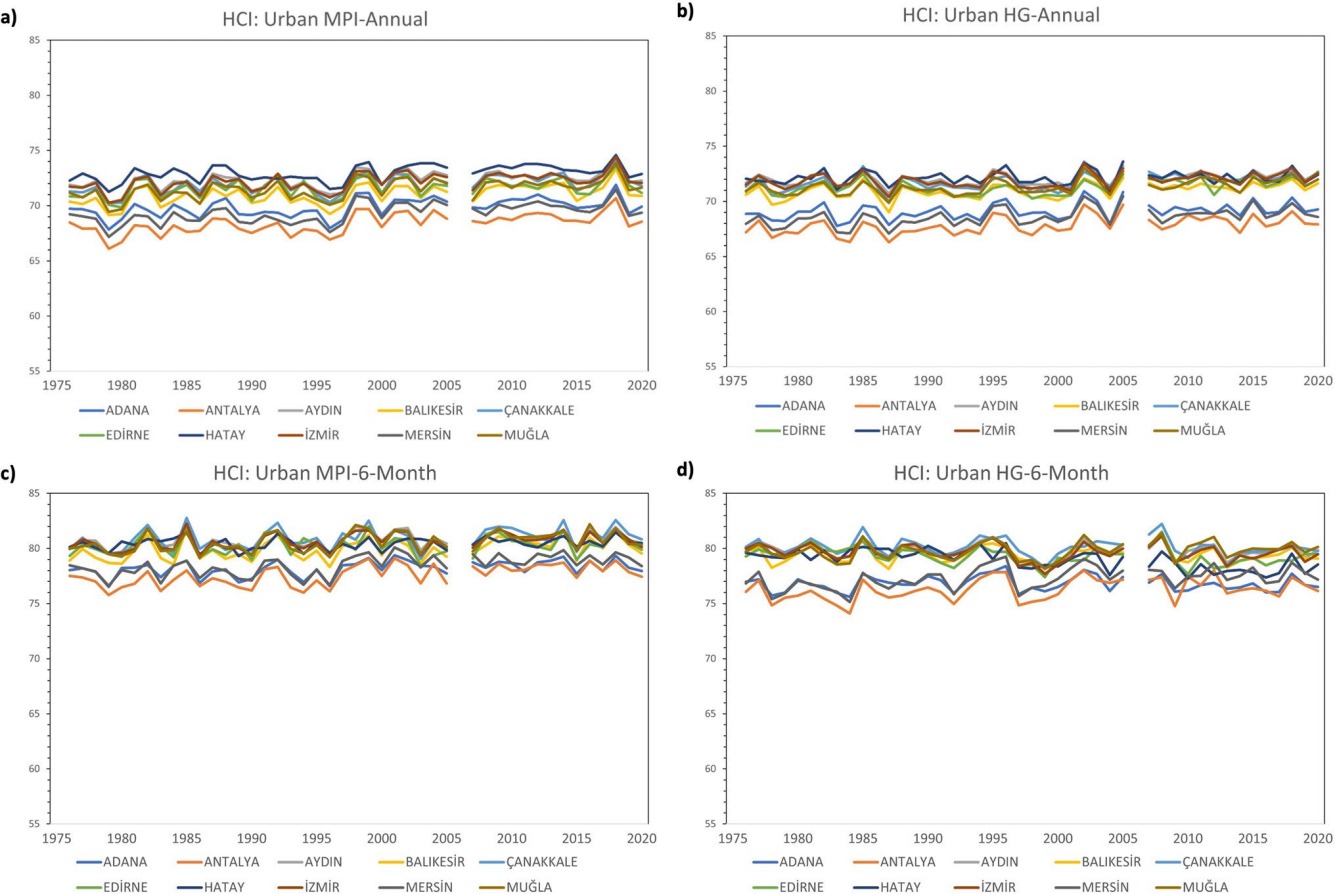
Trend analysis for HCI: Urban **a**) MPI-Annual **b**) HG-Annual **c**) MPI-6-Month **d**) HG-6-Month

**Fig. 3:**
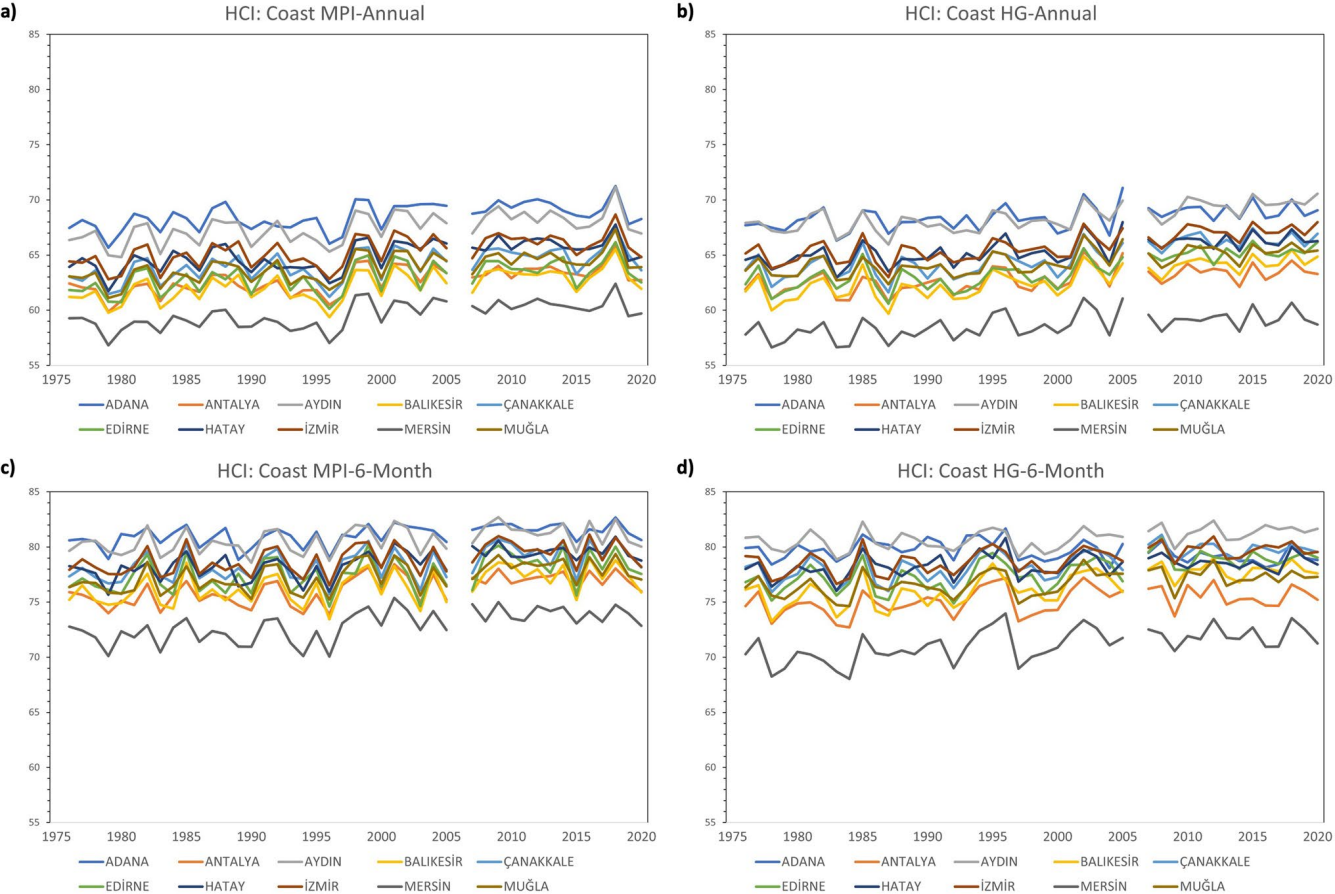
Trend analysis for HCI: Coast **a**) MPI-Annual **b**) HG-Annual **c**) MPI-6-Month **d**) HG-6-Month

**Fig. 4:**
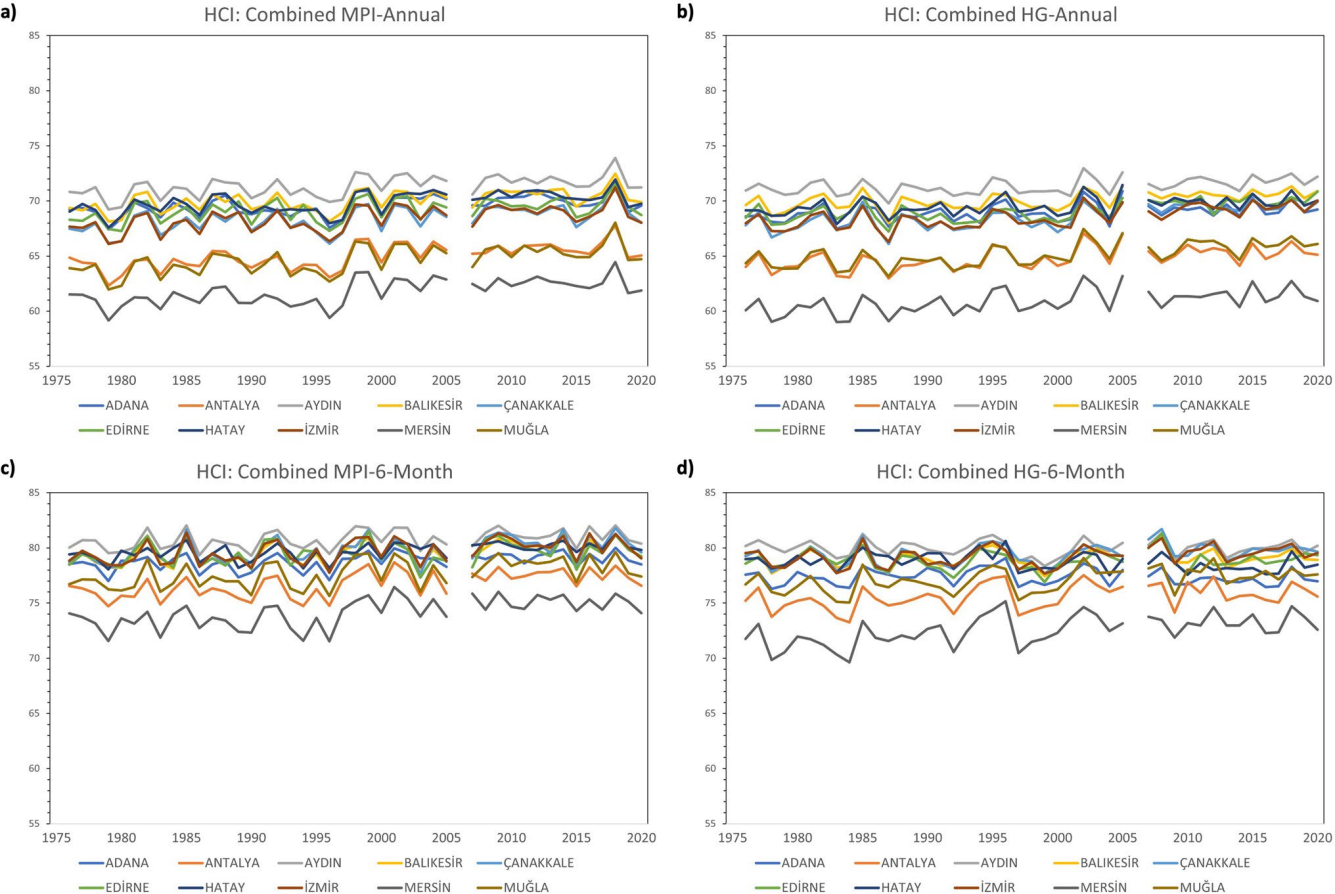
Trend analysis for HCI: Combined **a**) MPI-Annual **b**) HG-Annual **c**) MPI-6-Month **d**) HG-6-Month

The trend of the comfort conditions for HCI: Urban between 1976–2020 shows lower comfort conditions in Adana, Mersin, and Antalya in both annual and 6-month analyses. In the 6-month analyses, comfort scores are generally higher when all of the MPI-Annual, MPI-6-Month, HG-Annual, and HG-6-Month analyses are considered. When looking at the results in regard to HCI: Coast, it is seen that Mersin has the lowest comfort score. In both annual and 6-month analyses, it is seen that the best comfort conditions are in Adana and Aydın. However, MPI-Annual and HG-Annual analyses show that the difference between these two months and the other months is greater. As in HCI: Urban, comfort scores in 6-month analyses, including May–October, are generally higher when looking at all analyses of MPI-Annual, MPI-6-Month, HG-Annual, and HG-6-Month. When HCI: Combined is concerned, it is seen that the province with the least comfort conditions is Mersin. In MPI-Annual and HG-Annual analyses, it is seen that Antalya and Muğla have lower scores than other provinces. In the MPI-6-Month and HG-6-Month analyses, the scores of these provinces were closer to the other provinces (Figs. 2, 3 and 4).

In general, since there was no noticeable decrease in comfort conditions between 1976 and 2020, the first hypothesis of the study, “Comfort levels are expected to decrease from the past to the present,” was not fully confirmed. In the 6-month analyses performed for HCI: Urban, HCI: Coast and HCI: Combined in Aydın, Balıkesir, Çanakkale, Hatay and İzmir, decreases of 1–2 points were noticed between 1976 and 2020. Table 2

**Table 2:** The ratings of thermal comfort (based on humidex), aesthetic (based on cloud cover), precipitation, wind, and HCI: Urban scores

Thermal Comfort (TC)		Aesthetic (A)		Precipitation		Wind		HCI: Urban Score	
Humidex		Cloud Cover							
	Rate	%	Rate	mm	Rate	km/h	Rate		Rate
(-∞, -6) [-6, 0) [0, 7) [7, 11) [11, 15) [15, 18) [18, 20) [20, 23) [23, 26) [26, 27) [27, 29) [29, 31) [31, 33) [33, 35) [35, 37) [37, 39) [39, ∞)	1 2 3 4 5 6 7 9 10 9 8 7 6 5 4 2 0	[0, 1) [1, 11) [11, 21) [21, 31) [31, 41) [41, 51) [51, 61) [61, 71) [71, 81) [81, 91) [91, 100) 100	8 9 10 9 8 7 6 5 4 3 2 1	[0, 0.01) [0.01, 3) [3, 6) [6, 9) [9, 12) [12, 25) [25, ∞)	10 9 8 5 2 0 -1	[0, 0.01) [0.01, 10) [10, 20) [20, 30) [30, 40) [40, 50) [50, 70) [70, ∞)	8 10 9 8 6 3 0 -10	[0, 20) [20, 40) [40, 50) [50, 60) [60, 70) [70, 80) [80, 90) [90, 100)	Dangerous Unacceptable Marginal Acceptable Good Very Good Excellent Ideal

^*^ adapted from Tang (2013); Scott et al. (2016); Rutty et al. (2020);Demiroglu et al. (2020)

### Results of the panel data analysis

The findings derived from the study of panel data are presented (Table 3). Each row in the table shows six regression equations, where the dependent variable is either arrivals or overnight stays for the analysis of coastal, urban regions, and the whole province, respectively. In each equation, there is only one independent variable that represents the HCI (urban, coast, or combined). As such, each regression analyses the impact of holiday comfort on the number of tourist arrivals and overnight stays by doing separate regressions for each specification of the HCI (namely, MPI-6-month, MPI-annual, HG-6-month, and HG-annual) used as the independent variable.

**Table 3:** Results of the panel fixed effects analysis for arrivals and overnight stays

	HCI: Coast		HCI: Urban		HCI: Combined	
	Arrivals	Overnight Stays	Arrivals	Overnight Stays	Arrivals	Overnight Stays
MPI-6-month	0.393414*** (0.0724480)	0.291711*** (0.0771619)	0.457794*** (0.120510)	0.357312** (0.121140)	0.454280*** (0.0887818)	0.265394*** (0.0705967)
MPI-annual	0.495255*** (0.0728887)	0.369180*** (0.0796245)	0.650364*** (0.103466)	0.486967*** (0.115467)	0.588650*** (0.0833335)	0.351897*** (0.0799702)
HG-6-month	0.399214*** (0.0648793)	0.285411*** (0.0693395)	0.0201439 (0.164345)	0.0249142 (0.144356)	0.284532** (0.108336)	0.176227* (0.0879703)
HG-annual	0.295060*** (0.0357107)	0.209762*** (0.0395575)	0.299709*** (0.0650180)	0.216766*** (0.0651156)	0.324008*** (0.0461290)	0.162905*** (0.0471146)

^*^ Standard errors in parentheses. Detailed regression outputs are presented in Supplementary Material (Tables S1-S12)

#### Arrivals

The findings of HCI: Urban analysis indicate that the variables HG-annual, MPI-6-month, and MPI-annual have a positive impact on the influx of tourists in the metropolitan areas of the chosen regions. Nevertheless, the statistical analysis reveals that the coefficient associated with the HG-6-month variable is not statistically significant. The findings indicate that the comfort factor markedly influences the number of tourists going to the urban areas of the coastal provinces, implying that better climatic conditions and related comfort attract more tourists. It is evident that the MPI-annual variable exhibits greater importance compared to the other comfort variables, as indicated by its higher coefficient.

The findings of the HCI: Coast analysis indicate that the comfort variables, specifically referred to as MPI-6-month, and MPI-annual, HG-6-month, and HG-annual in the present study, have a positive effect on the number of visitors to the coastal locations under investigation. These findings show that climatic conditions considerably affect the level of comfort experienced by tourists. Furthermore, the level of comfort plays a crucial role in attracting tourists to the coastal regions of the provinces in the study. The coefficient associated with the MPI-annual variable is much higher compared to the coefficients of the other comfort variables. It implies that an increase in the MPI-annual level has a more pronounced impact on the number of visitors relative to the other comfort factors.

The findings of the analysis indicate a positive relationship between the variables MPI-6-month, MPI-annual, and HG-annual, and the number of tourists at the province level. This finding demonstrates the significance of the comfort factor in attracting tourists to the provinces under investigation. Specifically, when the level of comfort improves, there is an increase in the number of tourists visiting these regions. As in the case of HCI: Coast and HCI: Urban, it is observed that MPI-annual has a greater impact on the number of tourists due to its higher coefficient compared to those of the other variables.

#### Overnight stays

The findings of the analysis indicate that the variables MPI-6-month, MPI-annual, HG-6-month, and HG-annual have a positive effect on the number of overnight stays made by tourists in the coastal regions of the chosen provinces. In addition, climatic conditions influence the level of comfort experienced by tourists. The level of comfort has a substantial and positive impact on the duration of their stay in the provinces under investigation. The effectiveness of the MPI-annual variable is observed to be superior to that of the other comfort variables.

The findings of the analysis indicate that the variables HG-annual, MPI-6-month, and MPI-annual are positively associated with an increase in the number of overnight stays of tourists in urban areas. The comfort element considerably influences the duration of tourists’ visits to these provinces. Nevertheless, the coefficient of the HG-6-month variable lacks statistical significance, indicating that there is no discernible impact of HG-6-month on the number of overnight stays. The MPI-annual variable appears to have a greater impact in comparison to the other variables.

The findings of the analysis indicate that there is a positive relationship between the MPI-6-month, MPI-annual, and HG-annual components and the number of overnight stays by tourists at the province level. Climatic conditions notably influence the level of comfort experienced by tourists. Moreover, the level of comfort has a substantial impact on the duration of their vacation to the provinces under investigation. The marginal impact of the MPI-annual variable in increasing overnight stays is observed to be higher compared to the other variables.

### Results for future HCI projections for the 10 provinces

Findings on future HCI trends of ten provinces are presented in time series. Red and green lines represent trend graphs showing projections for MPI-ESM-MR and HadGEM2-ES, respectively. 1976–2020 is shown on the left axis and 2026–2050 is shown on the right axis. The analysis focused on the time frame spanning from 1976 to 2020, aiming to examine the trend in the number of tourist arrivals and overnight stays up until the year 2020.

**Fig. 5:**
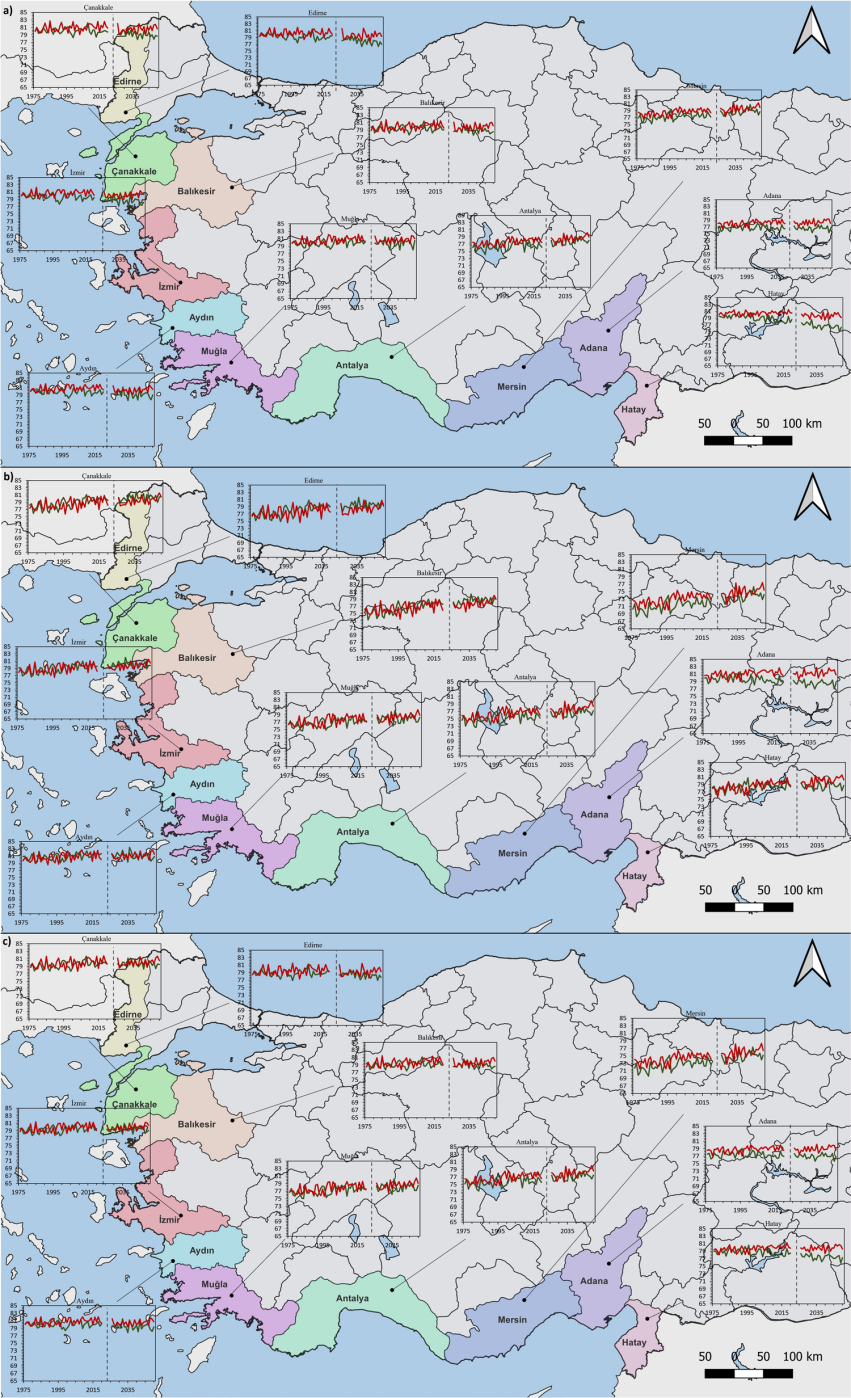
6-Month time series of **a**) HCI: Urban **b**) HCI: Coast **c**) HCI: Combined for the MPI-ESM-MR and HadGEM2-ES

When the annual trend of 6-month HCI: Urban is examined, HadGEM2-ES predicts lower HCI values than MPI-ESM-MR. For Çanakkale and İzmir, HadGEM2-ES HCI values are expected to decrease by approximately 1 point and MPI-ESM-MR by 0.5 points. For Edirne, HadGEM2-ES and MPI-ESM-MR predict a decrease of 1.5 points and 1 point, respectively. For Balıkesir, both models predict a slight decrease in HCI values. A very small increase is expected for Muğla. While a 0.5-point decrease is predicted in HadGEM2-ES and MPI-ESM-MR for Aydın, an increase is expected for Antalya and Mersin. HadGEM2-ES predicts a slight decrease for Adana, while MPI-ESM-MR predicts a slight increase. Both models predict a decrease in HCI values for Hatay. The analysis for the annual trend of the 6-month HCI means for HCI: Coast projects a 1-point decrease for HadGEM2-ES in Adana; however, a slight increase is expected for MPI-ESM-MR. For Çanakkale, Edirne, İzmir, and Balıkesir, an increase of 1 point for MPI-ESM-MR and 2 points for HadGEM2-ES is projected. A 1-point increase is expected for both MPI-ESM-MR and HadGEM2-ES in Muğla, Aydın, and Antalya. In contrast to other provinces, it is predicted that Mersin will have a 2-point increase in both MPI-ESM-MR and HadGEM2-ES. A slight increase for HadGEM2-ES and a 1-point increase for MPI-ESM-MR are projected for Hatay. When the annual trend of 6-month HCI: Combined is examined, HadGEM2-ES predicts lower HCI values than MPI-ESM-MR in several provinces. For Çanakkale and İzmir, both models project a slight increasing trend. HadGEM2-ES and MPI-ESM-MR project a slight downward trend for Edirne and a stable trend for Balıkesir. Both models show a 0.5-point decrease in Aydın and a 1-point increase in Muğla. For Antalya and Mersin, both models predict an increase of up to 2 points. Decreasing HCI values are noticeable in both HadGEM2-ES and MPI-ESM-MR models for Adana and Hatay (Fig. 5).

**Fig. 6:**
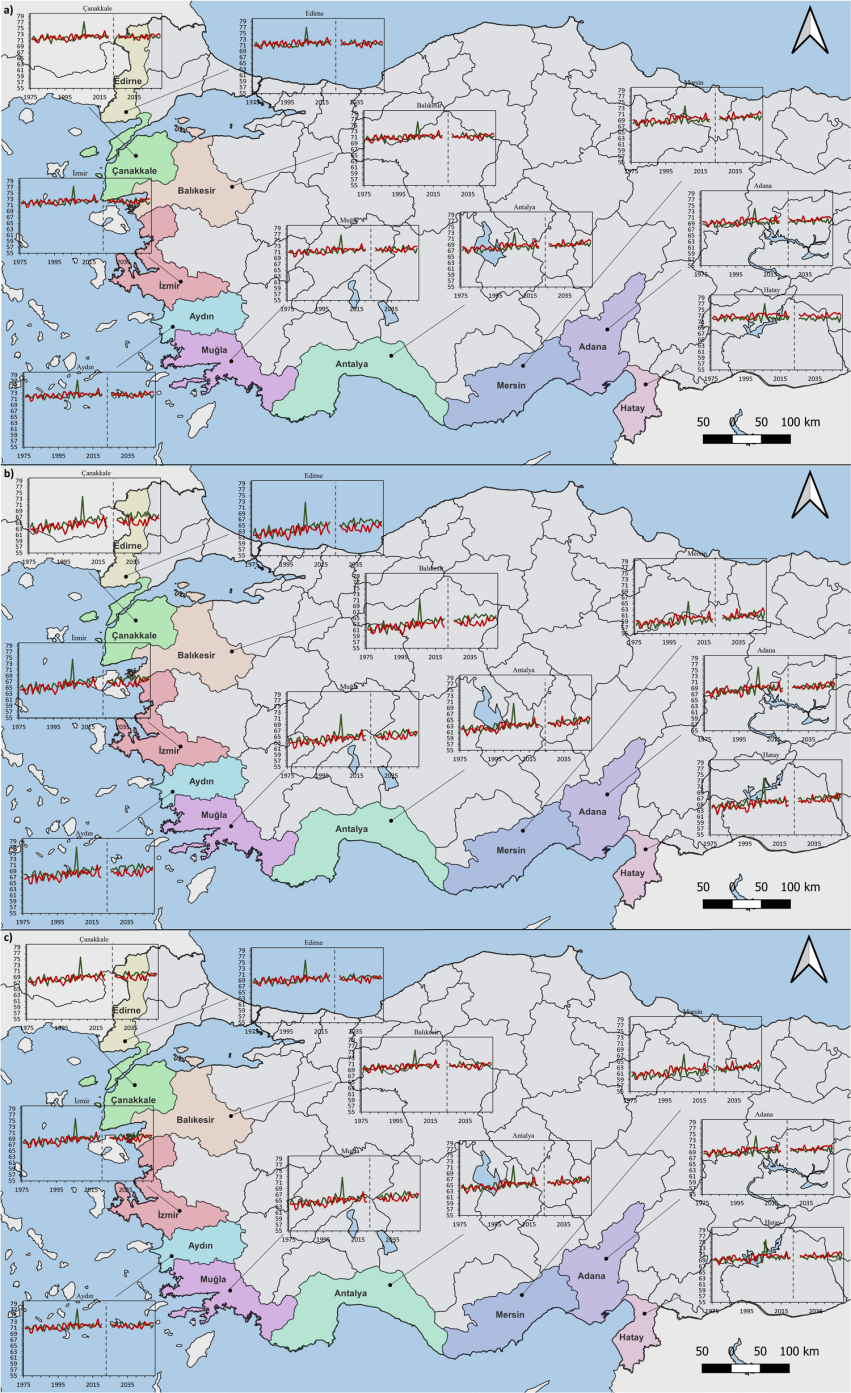
Annual time series of **a**) HCI: Urban **b**) HCI: Coast **c**) HCI: Combined for the MPI-ESM-MR and HadGEM2-ES

When HCI values related to the annual trend in the HCI: Urban calculation are examined, HadGEM2-ES projects a slight decrease and MPI-ESM-MR projects a 1-point increase for Adana. For Çanakkale, Edirne, İzmir, Balıkesir, Muğla, Aydın, a slight increase is expected in both MPI-ESM-MR and HadGEM2-ES. For Antalya and Mersin, MPI-ESM-MR, and HadGEM2-ES projects that HCI scores will increase. For Hatay, a slight decrease in HadGEM2-ES HCI values and an increase in MPI-ESM-MR are expected. When the annual trend of HCI: Coast is examined, both HadGEM2-ES and MPI-ESM-MR show a 1-point increase for Adana. An increase of 1 point in MPI-ESM-MR and 2 points in HadGEM2-ES is predicted for Çanakkale. For Edirne, MPI-ESM-MR shows an increase of 1 point, while HadGEM2-ES shows an increase of 3 points. For Izmir, both MPI-ESM-MR and HadGEM2-ES predict a 1-point increase. For Balıkesir, Aydın, and Hatay, MPI-ESM-MR predicts a 1-point and HadGEM2-ES a 2-point increase. A 2-point increase is expected in both models for Muğla, Antalya, and Mersin. When the trend of annual HCI values is examined, HadGEM2-ES and MPI-ESM-MR models show similar values in the HCI: Combined calculation. For Edirne, Çanakkale, Balıkesir, İzmir, and Aydın, HCI shows an increasing trend over time in both models. For Muğla, Antalya, Mersin, Adana, and Hatay, both models project increases of up to 2.5 points. Increases in annual means indicate that periods that were previously unfavourable or less favourable in terms of tourism will become more favourable in the future (Fig. 6).

## Discussion

Based on the findings of the HCI: Coast analysis, which examines the coastal districts, it is evident that the comfort variables have a positive effect on both the number of visitors to the coastal areas of the selected regions and the duration of their stays in these places. A similar outcome was obtained in the case of HCI: Urban, with the exception of the HG-6-month analysis. This finding demonstrates that comfort variables are also effective in activities such as walking. The HCI: Combined analysis reveals that the comfort variables have a positive effect on both the number of tourists visiting the selected provinces and the number of overnight stays in these places, when considering the provincial average. The research findings validate existing literature studies that investigate the impacts of climate change on tourist comfort. These studies have identified favorable associations between arrival statistics and indices measuring tourist comfort (Demiroglu et al. 2020; Aygün Oğur and Baycan 2023). Previous research in the literature has provided evidence of a robust correlation between tourism indices and the number of overnight stays in various destinations (Moreno and Amelung 2009; Rosselló-Nadal 2014).

If the current climate projections of the IPCC are accurate, popular destinations in the Mediterranean will experience a decline in attractiveness by the 2050s that can be attributed to the excessively high temperatures during the summer months, which coincide with peak holiday activity. Consequently, the optimal duration for vacationing in these destinations is expected to decrease. Furthermore, climate change scenarios indicate potential alterations, including the Mediterranean region experiencing a more favourable environment during the spring and autumn seasons (Amelung and Viner 2006). A study that examines the relationship between changes in climate comfort levels and the number of international tourists using the TCI and a regression model suggests that climate change will likely lead to significant declines in tourism demand, seasonal shifts, and the emergence of alternative destinations (Aygün Oğur and Baycan 2023).

The appropriateness of adaptation as a strategy for mitigating the tourism industry’s vulnerability to climate change has been acknowledged. The necessity of adapting to climate change is well recognized; however, it is arguable that the tourism industry lacks a comprehensive understanding of this requirement. Focusing on adaption techniques may prove to be a more straightforward and efficient approach once the potential impacts of climate change are comprehended (Dubois and Ceron 2006). The distinction between an impact and an adaptation study lies in this criterion. The assessment and understanding of vulnerabilities and corresponding adaptation solutions necessitate a comprehensive examination of the impacts of climate change on a given system (IPCC 2022). The analysis of the impacts of climate change on a particular system necessitates a shift towards the assessment and understanding of the system’s vulnerabilities and the corresponding strategies for adaptation.

Adaptation to climate change aims to ensure that the strategies implemented mitigate, cope with, and benefit from climate events. A sustainable approach to tourism destinations requires having a long-term perspective. Tourism destinations must respond to climate change through adaptation in terms of tourist facilities and services (Fennell and Cooper 2020). The tourism sector can adopt some strategies to combat climate change and ensure sustainable tourism (Mooney et al. 2009): It can balance the carbon emissions created by tourism. It can reduce tourism’s impact on climate change by changing industry practices and consumer behaviour. Mitigation can be achieved through behavioural (e.g., changing travel patterns), technological (e.g., the movement towards electric vehicles), and policy approaches (Hopkins and Higham 2018). Tourism destinations and consumer behaviour can be adapted to climate change. Collaboration with climate scientists can be undertaken to increase understanding of the links between carbon emissions in tourism, climate change, and societal needs and adaptation. There is an increasing body of literature that aims to forecast changes in travel patterns caused by climate change. The purpose of these publications is to provide guidance to the travel industry in terms of planning future operations and adapting to evolving climatic conditions (Gössling and Hall 2006). The adaptation strategies that are most commonly cited are efforts to diversify products or change destinations. The proposal of diversifying tourism products has been put forth as a potential mechanism for adaptation, and it is widely acknowledged as an effective strategy for reducing vulnerability to economic and other crises (Dubois and Ceron 2006; Gómez-Martín et al. 2014). Considering the risks involved with climate change, it is arguable that implementing planned adaptation strategies may be the most reasonable approach. However, it is essential for the planned adaptation process to incorporate various components such as risk management planning, financing of the adaptation process, prioritization of research and development initiatives, education, and effective communication, as well as assuming accountabilities for all stakeholders, ranging from residents to governmental entities.

This research has certain limitations. The Ministry of Culture and Tourism provides data on tourist arrivals and overnight stays dating back to 1976. Hence, there is a lack of data for the pre-1976 period. The study acknowledges the absence of reliable data regarding tourism activities in the selected provinces, except for the number of tourists and overnight stays. A more thorough examination of historical and prospective data may have been achieved by incorporating more indicators related to tourism. The study also provides recommendations for further investigation. The research in the literature sometimes lacks the inclusion of regional analyses pertaining to climate change, holiday comfort, and tourism activities. The primary objective of this study is to address the existing knowledge gap within this particular domain. However, there is a need to enhance the scope of impact studies to prioritize research that is centred around adaptability in this particular field. There should be a greater emphasis on conducting additional validation procedures to assess tourist performance measures, such as visitor satisfaction. Given the significance of short-term planning within the tourism industry, an analysis has been conducted to assess the near-future prospects. However, it is also possible to examine the long-term implications, such as those extending to the end of the century. The findings are expected to have significant implications for many individuals and entities involved in the tourism industry, including tourists, tourism professionals, operators, stakeholders, and policymakers.

## Conclusion

This study examines the annual and 6-month analyses of the HCI indices, specifically HCI: Coast, HCI: Urban, and HCI: Combined, utilizing data from 10 provinces (Adana, Antalya, Aydın, Balıkesir, Çanakkale, Edirne, Hatay, İzmir, Mersin, Muğla) between the years 1976 and 2020. It employs the panel fixed effects model to conduct an econometric analysis of the impact of comfort on tourist arrivals and overnight stays. Tourism, a sector deeply intertwined with environmental conditions, faces significant challenges due to climate change, which directly affects tourists' comfort levels. As climate patterns change and extreme weather events become more frequent, the comfort and safety of tourists are increasingly compromised. Rising temperatures, unpredictable weather conditions and environmental degradation can lead to discomfort and dissatisfaction for tourists. As a result, there appears to be a potential reduction in tourist numbers and overnight stays as a result of worsening comfort levels caused by climate change. As tourists prioritize destinations with suitable climates and enjoyable experiences, destinations facing negative climate impacts may experience a decrease in the number of visitors. Implementing measures to combat climate change and increase tourist comfort is essential to ensure the sustainability of the tourism industry and its resilience in the face of environmental challenges.

## Supplementary Information

Below is the link to the electronic supplementary material.

Supplementary file1 (DOCX 78 KB)

## Data Availability

The datasets are available from the corresponding author upon reasonable request.
