# Supplementary material for: A synthetic approach to the Holiday Climate Index for the Mediterranean Coast of Türkiye
Source: Int J Biometeorol. 2024 Jun 5;68(9):1773–87. doi: 10.1007/s00484-024-02704-7 (PMC11461600; doi:10.1007/s00484-024-02704-7)
Supplement: Supplementary file 1 — Supplementary file1 (DOCX 78 KB) [file 484_2024_2704_MOESM1_ESM.docx]

**TOURIST ARRIVALS DATA**

**Table S1.** HadGEM2 6-Month and Annual Analysis for HCI:Coast

Model 4: Fixed-effects, using 415 observations

Included 10 cross-sectional units

Time-series length: minimum 40, maximum 44

Dependent variable: l_Touristarrivals

Robust (HAC) standard errors

|  | *Coefficient* | *Std. Error* | *t-ratio* | *p-value* |  |
| --- | --- | --- | --- | --- | --- |
| const | −17.9847 | 5.02786 | −3.577 | 0.0060 | *** |
| HCI_HG_6month | 0.399214 | 0.0648793 | 6.153 | 0.0002 | *** |

| Mean dependent var | 12.95269 | S.D. dependent var | 1.477727 |
| --- | --- | --- | --- |
| Sum squared resid | 531.0335 | S.E. of regression | 1.146490 |
| LSDV R-squared | 0.412601 | Within R-squared | 0.149966 |
| Log-likelihood | −640.0179 | Akaike criterion | 1302.036 |
| Schwarz criterion | 1346.347 | Hannan-Quinn | 1319.558 |
| rho | 0.789223 | Durbin-Watson | 0.365344 |

Joint test on named regressors -

Test statistic: F(1, 9) = 37.8616

with p-value = P(F(1, 9) > 37.8616) = 0.000168075

Robust test for differing group intercepts -

Null hypothesis: The groups have a common intercept Test statistic: Welch F(9, 164.8) = 21.3034

with p-value = P(F(9, 164.8) > 21.3034) = 1.38163e-23

Model 2: Fixed-effects, using 415 observations

Included 10 cross-sectional units

Time-series length: minimum 40, maximum 44

Dependent variable: l_Touristarrivals

Robust (HAC) standard errors

|  | *Coefficient* | *Std. Error* | *t-ratio* | *p-value* |  |
| --- | --- | --- | --- | --- | --- |
| const | −6.18600 | 2.31633 | −2.671 | 0.0256 | ** |
| HCI_HG_annual | 0.295060 | 0.0357107 | 8.262 | <0.0001 | *** |

| Mean dependent var | 12.95269 | S.D. dependent var | 1.477727 |
| --- | --- | --- | --- |
| Sum squared resid | 514.8303 | S.E. of regression | 1.128863 |
| LSDV R-squared | 0.430524 | Within R-squared | 0.175903 |
| Log-likelihood | −633.5880 | Akaike criterion | 1289.176 |
| Schwarz criterion | 1333.487 | Hannan-Quinn | 1306.698 |
| rho | 0.757812 | Durbin-Watson | 0.433560 |

Joint test on named regressors -

Test statistic: F(1, 9) = 68.2689

with p-value = P(F(1, 9) > 68.2689) = 1.70867e-05

Robust test for differing group intercepts -

Null hypothesis: The groups have a common intercept Test statistic: Welch F(9, 164.7) = 24.4824

with p-value = P(F(9, 164.7) > 24.4824) = 2.95865e-26

**Table S2.** MPI-ESM-MR 6-Month and Annual Analysis for HCI:Coast

Model 3: Fixed-effects, using 415 observations

Included 10 cross-sectional units

Time-series length: minimum 40, maximum 44

Dependent variable: l_Touristarrivals

Robust (HAC) standard errors

|  | *Coefficient* | *Std. Error* | *t-ratio* | *p-value* |  |
| --- | --- | --- | --- | --- | --- |
| const | −17.7113 | 5.64683 | −3.136 | 0.0120 | ** |
| HCI_MPI_6month | 0.393414 | 0.0724480 | 5.430 | 0.0004 | *** |

| Mean dependent var | 12.95269 | S.D. dependent var | 1.477727 |
| --- | --- | --- | --- |
| Sum squared resid | 520.0165 | S.E. of regression | 1.134535 |
| LSDV R-squared | 0.424788 | Within R-squared | 0.167601 |
| Log-likelihood | −635.6677 | Akaike criterion | 1293.335 |
| Schwarz criterion | 1337.647 | Hannan-Quinn | 1310.858 |
| rho | 0.710675 | Durbin-Watson | 0.522777 |

Joint test on named regressors -

Test statistic: F(1, 9) = 29.4881

with p-value = P(F(1, 9) > 29.4881) = 0.000416213

Robust test for differing group intercepts -

Null hypothesis: The groups have a common intercept Test statistic: Welch F(9, 164.8) = 22.6583

with p-value = P(F(9, 164.8) > 22.6583) = 9.52304e-25

Model 1: Fixed-effects, using 415 observations

Included 10 cross-sectional units

Time-series length: minimum 40, maximum 44

Dependent variable: l_Touristarrivals

Robust (HAC) standard errors

|  | *Coefficient* | *Std. Error* | *t-ratio* | *p-value* |  |
| --- | --- | --- | --- | --- | --- |
| const | −18.9360 | 4.69319 | −4.035 | 0.0030 | *** |
| HCI_MPI_annual | 0.495255 | 0.0728887 | 6.795 | <0.0001 | *** |

| Mean dependent var | 12.95269 | S.D. dependent var | 1.477727 |
| --- | --- | --- | --- |
| Sum squared resid | 470.9365 | S.E. of regression | 1.079669 |
| LSDV R-squared | 0.479077 | Within R-squared | 0.246165 |
| Log-likelihood | −615.0968 | Akaike criterion | 1252.194 |
| Schwarz criterion | 1296.505 | Hannan-Quinn | 1269.716 |
| rho | 0.658152 | Durbin-Watson | 0.608386 |

Joint test on named regressors -

Test statistic: F(1, 9) = 46.1677

with p-value = P(F(1, 9) > 46.1677) = 7.95295e-05

Robust test for differing group intercepts -

Null hypothesis: The groups have a common intercept Test statistic: Welch F(9, 164.7) = 24.1479

with p-value = P(F(9, 164.7) > 24.1479) = 5.53139e-26

**Table S3.** HadGEM2 6-Month and Annual Analysis for HCI:Urban

Model 4: Fixed-effects, using 415 observations

Included 10 cross-sectional units

Time-series length: minimum 40, maximum 44

Dependent variable: l_Touristarrivals

Robust (HAC) standard errors

|  | *Coefficient* | *Std. Error* | *t-ratio* | *p-value* |
| --- | --- | --- | --- | --- |
| const | 11.3658 | 12.9467 | 0.8779 | 0.4028 |
| HCI_HG_6month | 0.0201439 | 0.164345 | 0.1226 | 0.9051 |

| Mean dependent var | 12.95269 | S.D. dependent var | 1.477727 |
| --- | --- | --- | --- |
| Sum squared resid | 624.6053 | S.E. of regression | 1.243404 |
| LSDV R-squared | 0.309097 | Within R-squared | 0.000184 |
| Log-likelihood | −673.6941 | Akaike criterion | 1369.388 |
| Schwarz criterion | 1413.699 | Hannan-Quinn | 1386.910 |
| rho | 0.931917 | Durbin-Watson | 0.112935 |

Joint test on named regressors -

Test statistic: F(1, 9) = 0.0150236

with p-value = P(F(1, 9) > 0.0150236) = 0.90514

Robust test for differing group intercepts -

Null hypothesis: The groups have a common intercept Test statistic: Welch F(9, 164.7) = 21.8415

with p-value = P(F(9, 164.7) > 21.8415) = 4.73846e-24

Model 2: Fixed-effects, using 415 observations

Included 10 cross-sectional units

Time-series length: minimum 40, maximum 44

Dependent variable: l_Touristarrivals

Robust (HAC) standard errors

|  | *Coefficient* | *Std. Error* | *t-ratio* | *p-value* |  |
| --- | --- | --- | --- | --- | --- |
| const | −8.29093 | 4.60852 | −1.799 | 0.1056 |  |
| HCI_HG_annual | 0.299709 | 0.0650180 | 4.610 | 0.0013 | *** |

| Mean dependent var | 12.95269 | S.D. dependent var | 1.477727 |
| --- | --- | --- | --- |
| Sum squared resid | 582.6070 | S.E. of regression | 1.200873 |
| LSDV R-squared | 0.355554 | Within R-squared | 0.067412 |
| Log-likelihood | −659.2506 | Akaike criterion | 1340.501 |
| Schwarz criterion | 1384.812 | Hannan-Quinn | 1358.023 |
| rho | 0.863172 | Durbin-Watson | 0.240481 |

Joint test on named regressors -

Test statistic: F(1, 9) = 21.2488

with p-value = P(F(1, 9) > 21.2488) = 0.00127295

Robust test for differing group intercepts -

Null hypothesis: The groups have a common intercept Test statistic: Welch F(9, 164.7) = 19.924

with p-value = P(F(9, 164.7) > 19.924) = 2.30141e-22

**Table S4.** MPI-ESM-MR 6-Month and Annual Analysis for HCI:Urban

Model 3: Fixed-effects, using 415 observations

Included 10 cross-sectional units

Time-series length: minimum 40, maximum 44

Dependent variable: l_Touristarrivals

Robust (HAC) standard errors

|  | *Coefficient* | *Std. Error* | *t-ratio* | *p-value* |  |
| --- | --- | --- | --- | --- | --- |
| const | −23.5947 | 9.62076 | −2.452 | 0.0366 | ** |
| HCI_MPI_6month | 0.457794 | 0.120510 | 3.799 | 0.0042 | *** |

| Mean dependent var | 12.95269 | S.D. dependent var | 1.477727 |
| --- | --- | --- | --- |
| Sum squared resid | 565.7132 | S.E. of regression | 1.183334 |
| LSDV R-squared | 0.374240 | Within R-squared | 0.094454 |
| Log-likelihood | −653.1448 | Akaike criterion | 1328.290 |
| Schwarz criterion | 1372.601 | Hannan-Quinn | 1345.812 |
| rho | 0.807808 | Durbin-Watson | 0.338944 |

Joint test on named regressors -

Test statistic: F(1, 9) = 14.431

with p-value = P(F(1, 9) > 14.431) = 0.00422508

Robust test for differing group intercepts -

Null hypothesis: The groups have a common intercept Test statistic: Welch F(9, 164.7) = 20.7372

with p-value = P(F(9, 164.7) > 20.7372) = 4.33738e-23

Model 1: Fixed-effects, using 415 observations

Included 10 cross-sectional units

Time-series length: minimum 40, maximum 44

Dependent variable: l_Touristarrivals

Robust (HAC) standard errors

|  | *Coefficient* | *Std. Error* | *t-ratio* | *p-value* |  |
| --- | --- | --- | --- | --- | --- |
| const | −33.3584 | 7.36763 | −4.528 | 0.0014 | *** |
| HCI_MPI_annual | 0.650364 | 0.103466 | 6.286 | 0.0001 | *** |

| Mean dependent var | 12.95269 | S.D. dependent var | 1.477727 |
| --- | --- | --- | --- |
| Sum squared resid | 510.8005 | S.E. of regression | 1.124437 |
| LSDV R-squared | 0.434982 | Within R-squared | 0.182354 |
| Log-likelihood | −631.9574 | Akaike criterion | 1285.915 |
| Schwarz criterion | 1330.226 | Hannan-Quinn | 1303.437 |
| rho | 0.717265 | Durbin-Watson | 0.483677 |

Joint test on named regressors -

Test statistic: F(1, 9) = 39.5107

with p-value = P(F(1, 9) > 39.5107) = 0.000143392

Robust test for differing group intercepts -

Null hypothesis: The groups have a common intercept Test statistic: Welch F(9, 164.7) = 19.4372

with p-value = P(F(9, 164.7) > 19.4372) = 6.34917e-22

**Table S5.** HadGEM2 6-Month and Annual Analysis for HCI:Combined

Model 4: Fixed-effects, using 415 observations

Included 10 cross-sectional units

Time-series length: minimum 40, maximum 44

Dependent variable: l_Touristarrivals

Robust (HAC) standard errors

|  | *Coefficient* | *Std. Error* | *t-ratio* | *p-value* |  |
| --- | --- | --- | --- | --- | --- |
| const | −9.22500 | 8.43577 | −1.094 | 0.3026 |  |
| HCI_HG_6month | 0.284532 | 0.108336 | 2.626 | 0.0275 | ** |

| Mean dependent var | 12.93065 | S.D. dependent var | 1.469641 |
| --- | --- | --- | --- |
| Sum squared resid | 589.0887 | S.E. of regression | 1.207535 |
| LSDV R-squared | 0.341194 | Within R-squared | 0.046471 |
| Log-likelihood | −661.5464 | Akaike criterion | 1345.093 |
| Schwarz criterion | 1389.404 | Hannan-Quinn | 1362.615 |
| rho | 0.887800 | Durbin-Watson | 0.190018 |

Joint test on named regressors -

Test statistic: F(1, 9) = 6.89794

with p-value = P(F(1, 9) > 6.89794) = 0.027521

Robust test for differing group intercepts -

Null hypothesis: The groups have a common intercept Test statistic: Welch F(9, 164.7) = 19.86

with p-value = P(F(9, 164.7) > 19.86) = 2.63053e-22

Model 2: Fixed-effects, using 415 observations

Included 10 cross-sectional units

Time-series length: minimum 40, maximum 44

Dependent variable: l_Touristarrivals

Robust (HAC) standard errors

|  | *Coefficient* | *Std. Error* | *t-ratio* | *p-value* |  |
| --- | --- | --- | --- | --- | --- |
| const | −9.07709 | 3.13324 | −2.897 | 0.0177 | ** |
| HCI_HG_annual | 0.324008 | 0.0461290 | 7.024 | <0.0001 | *** |

| Mean dependent var | 12.93065 | S.D. dependent var | 1.469641 |
| --- | --- | --- | --- |
| Sum squared resid | 539.9632 | S.E. of regression | 1.156089 |
| LSDV R-squared | 0.396133 | Within R-squared | 0.125988 |
| Log-likelihood | −643.4782 | Akaike criterion | 1308.956 |
| Schwarz criterion | 1353.267 | Hannan-Quinn | 1326.478 |
| rho | 0.805063 | Durbin-Watson | 0.349014 |

Joint test on named regressors -

Test statistic: F(1, 9) = 49.336

with p-value = P(F(1, 9) > 49.336) = 6.15985e-05

Robust test for differing group intercepts -

Null hypothesis: The groups have a common intercept Test statistic: Welch F(9, 164.7) = 18.8153

with p-value = P(F(9, 164.7) > 18.8153) = 2.36339e-21

**Table S6.** MPI-ESM-MR 6-Month and Annual Analysis for HCI:Combined

Model 3: Fixed-effects, using 415 observations

Included 10 cross-sectional units

Time-series length: minimum 40, maximum 44

Dependent variable: l_Touristarrivals

Robust (HAC) standard errors

|  | *Coefficient* | *Std. Error* | *t-ratio* | *p-value* |  |
| --- | --- | --- | --- | --- | --- |
| const | −22.8273 | 6.98834 | −3.266 | 0.0097 | *** |
| HCI_MPI_6month | 0.454280 | 0.0887818 | 5.117 | 0.0006 | *** |

| Mean dependent var | 12.93065 | S.D. dependent var | 1.469641 |
| --- | --- | --- | --- |
| Sum squared resid | 530.1182 | S.E. of regression | 1.145502 |
| LSDV R-squared | 0.407143 | Within R-squared | 0.141924 |
| Log-likelihood | −639.6600 | Akaike criterion | 1301.320 |
| Schwarz criterion | 1345.631 | Hannan-Quinn | 1318.842 |
| rho | 0.753380 | Durbin-Watson | 0.443442 |

Joint test on named regressors -

Test statistic: F(1, 9) = 26.1817

with p-value = P(F(1, 9) > 26.1817) = 0.000630685

Robust test for differing group intercepts -

Null hypothesis: The groups have a common intercept Test statistic: Welch F(9, 164.7) = 20.3252

with p-value = P(F(9, 164.7) > 20.3252) = 1.00614e-22

Model 1: Fixed-effects, using 415 observations

Included 10 cross-sectional units

Time-series length: minimum 40, maximum 44

Dependent variable: l_Touristarrivals

Robust (HAC) standard errors

|  | *Coefficient* | *Std. Error* | *t-ratio* | *p-value* |  |
| --- | --- | --- | --- | --- | --- |
| const | −27.0586 | 5.66117 | −4.780 | 0.0010 | *** |
| HCI_MPI_annual | 0.588650 | 0.0833335 | 7.064 | <0.0001 | *** |

| Mean dependent var | 12.93065 | S.D. dependent var | 1.469641 |
| --- | --- | --- | --- |
| Sum squared resid | 478.5426 | S.E. of regression | 1.088353 |
| LSDV R-squared | 0.464823 | Within R-squared | 0.225406 |
| Log-likelihood | −618.4213 | Akaike criterion | 1258.843 |
| Schwarz criterion | 1303.154 | Hannan-Quinn | 1276.365 |
| rho | 0.685124 | Durbin-Watson | 0.551958 |

Joint test on named regressors -

Test statistic: F(1, 9) = 49.8971

with p-value = P(F(1, 9) > 49.8971) = 5.89605e-05

Robust test for differing group intercepts -

Null hypothesis: The groups have a common intercept Test statistic: Welch F(9, 164.7) = 17.8915

with p-value = P(F(9, 164.7) > 17.8915) = 1.72908e-20

**OVERNIGHT STAYS DATA**

**Table S7.** HadGEM2 6-Month and Annual Analysis for HCI:Coast

Model 8: Fixed-effects, using 415 observations

Included 10 cross-sectional units

Time-series length: minimum 40, maximum 44

Dependent variable: l_Overnightstay

Robust (HAC) standard errors

|  | *Coefficient* | *Std. Error* | *t-ratio* | *p-value* |  |
| --- | --- | --- | --- | --- | --- |
| const | −8.60449 | 5.37351 | −1.601 | 0.1438 |  |
| HCI_HG_6month | 0.285411 | 0.0693395 | 4.116 | 0.0026 | *** |

| Mean dependent var | 13.51366 | S.D. dependent var | 1.674569 |
| --- | --- | --- | --- |
| Sum squared resid | 410.3196 | S.E. of regression | 1.007791 |
| LSDV R-squared | 0.646560 | Within R-squared | 0.104508 |
| Log-likelihood | −586.5060 | Akaike criterion | 1195.012 |
| Schwarz criterion | 1239.323 | Hannan-Quinn | 1212.534 |
| rho | 0.798289 | Durbin-Watson | 0.325639 |

Joint test on named regressors -

Test statistic: F(1, 9) = 16.9426

with p-value = P(F(1, 9) > 16.9426) = 0.00261293

Robust test for differing group intercepts -

Null hypothesis: The groups have a common intercept Test statistic: Welch F(9, 164.5) = 69.175

with p-value = P(F(9, 164.5) > 69.175) = 2.52659e-51

Model 6: Fixed-effects, using 415 observations

Included 10 cross-sectional units

Time-series length: minimum 40, maximum 44

Dependent variable: l_Overnightstay

Robust (HAC) standard errors

|  | *Coefficient* | *Std. Error* | *t-ratio* | *p-value* |  |
| --- | --- | --- | --- | --- | --- |
| const | −0.0922808 | 2.56585 | −0.03597 | 0.9721 |  |
| HCI_HG_annual | 0.209762 | 0.0395575 | 5.303 | 0.0005 | *** |

| Mean dependent var | 13.51366 | S.D. dependent var | 1.674569 |
| --- | --- | --- | --- |
| Sum squared resid | 402.6677 | S.E. of regression | 0.998350 |
| LSDV R-squared | 0.653151 | Within R-squared | 0.121208 |
| Log-likelihood | −582.5999 | Akaike criterion | 1187.200 |
| Schwarz criterion | 1231.511 | Hannan-Quinn | 1204.722 |
| rho | 0.777368 | Durbin-Watson | 0.373127 |

Joint test on named regressors -

Test statistic: F(1, 9) = 28.1187

with p-value = P(F(1, 9) > 28.1187) = 0.000492094

Robust test for differing group intercepts -

Null hypothesis: The groups have a common intercept Test statistic: Welch F(9, 164.5) = 77.7379

with p-value = P(F(9, 164.5) > 77.7379) = 1.28109e-54

**Table S8.** MPI-ESM-MR 6-Month and Annual Analysis for HCI:Coast

Model 7: Fixed-effects, using 415 observations

Included 10 cross-sectional units

Time-series length: minimum 40, maximum 44

Dependent variable: l_Overnightstay

Robust (HAC) standard errors

|  | *Coefficient* | *Std. Error* | *t-ratio* | *p-value* |  |
| --- | --- | --- | --- | --- | --- |
| const | −9.22322 | 6.01425 | −1.534 | 0.1595 |  |
| HCI_MPI_6month | 0.291711 | 0.0771619 | 3.780 | 0.0043 | *** |

| Mean dependent var | 13.51366 | S.D. dependent var | 1.674569 |
| --- | --- | --- | --- |
| Sum squared resid | 400.6395 | S.E. of regression | 0.995832 |
| LSDV R-squared | 0.654898 | Within R-squared | 0.125634 |
| Log-likelihood | −581.5521 | Akaike criterion | 1185.104 |
| Schwarz criterion | 1229.415 | Hannan-Quinn | 1202.626 |
| rho | 0.745875 | Durbin-Watson | 0.429134 |

Joint test on named regressors -

Test statistic: F(1, 9) = 14.2922

with p-value = P(F(1, 9) > 14.2922) = 0.00434568

Robust test for differing group intercepts -

Null hypothesis: The groups have a common intercept Test statistic: Welch F(9, 164.5) = 72.3952

with p-value = P(F(9, 164.5) > 72.3952) = 1.34426e-52

Model 5: Fixed-effects, using 415 observations

Included 10 cross-sectional units

Time-series length: minimum 40, maximum 44

Dependent variable: l_Overnightstay

Robust (HAC) standard errors

|  | *Coefficient* | *Std. Error* | *t-ratio* | *p-value* |  |
| --- | --- | --- | --- | --- | --- |
| const | −10.2573 | 5.12690 | −2.001 | 0.0765 | * |
| HCI_MPI_annual | 0.369180 | 0.0796245 | 4.637 | 0.0012 | *** |

| Mean dependent var | 13.51366 | S.D. dependent var | 1.674569 |
| --- | --- | --- | --- |
| Sum squared resid | 372.7524 | S.E. of regression | 0.960549 |
| LSDV R-squared | 0.678919 | Within R-squared | 0.186496 |
| Log-likelihood | −566.5814 | Akaike criterion | 1155.163 |
| Schwarz criterion | 1199.474 | Hannan-Quinn | 1172.685 |
| rho | 0.707783 | Durbin-Watson | 0.486672 |

Joint test on named regressors -

Test statistic: F(1, 9) = 21.4973

with p-value = P(F(1, 9) > 21.4973) = 0.0012253

Robust test for differing group intercepts -

Null hypothesis: The groups have a common intercept Test statistic: Welch F(9, 164.5) = 77.4095

with p-value = P(F(9, 164.5) > 77.4095) = 1.69868e-54

**Table S9.** HadGEM2 6-Month and Annual Analysis for HCI:Urban

Model 8: Fixed-effects, using 415 observations

Included 10 cross-sectional units

Time-series length: minimum 40, maximum 44

Dependent variable: l_Overnightstay

Robust (HAC) standard errors

|  | *Coefficient* | *Std. Error* | *t-ratio* | *p-value* |
| --- | --- | --- | --- | --- |
| const | 11.5510 | 11.3721 | 1.016 | 0.3363 |
| HCI_HG_6month | 0.0249142 | 0.144356 | 0.1726 | 0.8668 |

| Mean dependent var | 13.51366 | S.D. dependent var | 1.674569 |
| --- | --- | --- | --- |
| Sum squared resid | 458.0296 | S.E. of regression | 1.064771 |
| LSDV R-squared | 0.605463 | Within R-squared | 0.000385 |
| Log-likelihood | −609.3305 | Akaike criterion | 1240.661 |
| Schwarz criterion | 1284.972 | Hannan-Quinn | 1258.183 |
| rho | 0.896598 | Durbin-Watson | 0.154896 |

Joint test on named regressors -

Test statistic: F(1, 9) = 0.0297866

with p-value = P(F(1, 9) > 0.0297866) = 0.866794

Robust test for differing group intercepts -

Null hypothesis: The groups have a common intercept Test statistic: Welch F(9, 164.5) = 77.2005

with p-value = P(F(9, 164.5) > 77.2005) = 1.99618e-54

Model 6: Fixed-effects, using 415 observations

Included 10 cross-sectional units

Time-series length: minimum 40, maximum 44

Dependent variable: l_Overnightstay

Robust (HAC) standard errors

|  | *Coefficient* | *Std. Error* | *t-ratio* | *p-value* |  |
| --- | --- | --- | --- | --- | --- |
| const | −1.85091 | 4.61544 | −0.4010 | 0.6978 |  |
| HCI_HG_annual | 0.216766 | 0.0651156 | 3.329 | 0.0088 | *** |

| Mean dependent var | 13.51366 | S.D. dependent var | 1.674569 |
| --- | --- | --- | --- |
| Sum squared resid | 436.1763 | S.E. of regression | 1.039059 |
| LSDV R-squared | 0.624287 | Within R-squared | 0.048078 |
| Log-likelihood | −599.1863 | Akaike criterion | 1220.373 |
| Schwarz criterion | 1264.684 | Hannan-Quinn | 1237.895 |
| rho | 0.843994 | Durbin-Watson | 0.252707 |

Joint test on named regressors -

Test statistic: F(1, 9) = 11.0819

with p-value = P(F(1, 9) > 11.0819) = 0.00881382

Robust test for differing group intercepts -

Null hypothesis: The groups have a common intercept Test statistic: Welch F(9, 164.6) = 63.0755

with p-value = P(F(9, 164.6) > 63.0755) = 8.90538e-49

**Table S10.** MPI-ESM-MR 6-Month and Annual Analysis for HCI:Urban

Model 7: Fixed-effects, using 415 observations

Included 10 cross-sectional units

Time-series length: minimum 40, maximum 44

Dependent variable: l_Overnightstay

Robust (HAC) standard errors

|  | *Coefficient* | *Std. Error* | *t-ratio* | *p-value* |  |
| --- | --- | --- | --- | --- | --- |
| const | −15.0119 | 9.67102 | −1.552 | 0.1550 |  |
| HCI_MPI_6month | 0.357312 | 0.121140 | 2.950 | 0.0162 | ** |

| Mean dependent var | 13.51366 | S.D. dependent var | 1.674569 |
| --- | --- | --- | --- |
| Sum squared resid | 422.2590 | S.E. of regression | 1.022348 |
| LSDV R-squared | 0.636275 | Within R-squared | 0.078451 |
| Log-likelihood | −592.4576 | Akaike criterion | 1206.915 |
| Schwarz criterion | 1251.226 | Hannan-Quinn | 1224.437 |
| rho | 0.799762 | Durbin-Watson | 0.326705 |

Joint test on named regressors -

Test statistic: F(1, 9) = 8.70009

with p-value = P(F(1, 9) > 8.70009) = 0.0162317

Robust test for differing group intercepts -

Null hypothesis: The groups have a common intercept Test statistic: Welch F(9, 164.6) = 66.4267

with p-value = P(F(9, 164.6) > 66.4267) = 3.37561e-50

Model 5: Fixed-effects, using 415 observations

Included 10 cross-sectional units

Time-series length: minimum 40, maximum 44

Dependent variable: l_Overnightstay

Robust (HAC) standard errors

|  | *Coefficient* | *Std. Error* | *t-ratio* | *p-value* |  |
| --- | --- | --- | --- | --- | --- |
| const | −21.1623 | 8.22217 | −2.574 | 0.0300 | ** |
| HCI_MPI_annual | 0.486967 | 0.115467 | 4.217 | 0.0022 | *** |

| Mean dependent var | 13.51366 | S.D. dependent var | 1.674569 |
| --- | --- | --- | --- |
| Sum squared resid | 394.3374 | S.E. of regression | 0.987969 |
| LSDV R-squared | 0.660326 | Within R-squared | 0.139388 |
| Log-likelihood | −578.2621 | Akaike criterion | 1178.524 |
| Schwarz criterion | 1222.835 | Hannan-Quinn | 1196.046 |
| rho | 0.740169 | Durbin-Watson | 0.413784 |

Joint test on named regressors -

Test statistic: F(1, 9) = 17.7862

with p-value = P(F(1, 9) > 17.7862) = 0.00224817

Robust test for differing group intercepts -

Null hypothesis: The groups have a common intercept Test statistic: Welch F(9, 164.6) = 57.3972

with p-value = P(F(9, 164.6) > 57.3972) = 3.15337e-46

**Table S11.** HadGEM2 6-Month and Annual Analysis for HCI:Combined

Model 9: Fixed-effects, using 315 observations

Included 10 cross-sectional units

Time-series length: minimum 17, maximum 44

Dependent variable: l_Overnightstay

Robust (HAC) standard errors

|  | *Coefficient* | *Std. Error* | *t-ratio* | *p-value* |  |
| --- | --- | --- | --- | --- | --- |
| const | −0.215368 | 6.84757 | −0.03145 | 0.9756 |  |
| HCI_HG_6month | 0.176227 | 0.0879703 | 2.003 | 0.0762 | * |

| Mean dependent var | 13.50204 | S.D. dependent var | 1.734971 |
| --- | --- | --- | --- |
| Sum squared resid | 204.4593 | S.E. of regression | 0.820100 |
| LSDV R-squared | 0.783682 | Within R-squared | 0.038962 |
| Log-likelihood | −378.8936 | Akaike criterion | 779.7871 |
| Schwarz criterion | 821.0654 | Hannan-Quinn | 796.2794 |
| rho | 0.857168 | Durbin-Watson | 0.252589 |

Joint test on named regressors -

Test statistic: F(1, 9) = 4.01302

with p-value = P(F(1, 9) > 4.01302) = 0.0761525

Robust test for differing group intercepts -

Null hypothesis: The groups have a common intercept Test statistic: Welch F(9, 106.9) = 403.07

with p-value = P(F(9, 106.9) > 403.07) = 3.25788e-78

Model 7: Fixed-effects, using 315 observations

Included 10 cross-sectional units

Time-series length: minimum 17, maximum 44

Dependent variable: l_Overnightstay

Robust (HAC) standard errors

|  | *Coefficient* | *Std. Error* | *t-ratio* | *p-value* |  |
| --- | --- | --- | --- | --- | --- |
| const | 2.46908 | 3.19090 | 0.7738 | 0.4589 |  |
| HCI_HG_annual | 0.162905 | 0.0471146 | 3.458 | 0.0072 | *** |

| Mean dependent var | 13.50204 | S.D. dependent var | 1.734971 |
| --- | --- | --- | --- |
| Sum squared resid | 195.8251 | S.E. of regression | 0.802597 |
| LSDV R-squared | 0.792817 | Within R-squared | 0.079546 |
| Log-likelihood | −372.0979 | Akaike criterion | 766.1958 |
| Schwarz criterion | 807.4741 | Hannan-Quinn | 782.6880 |
| rho | 0.809844 | Durbin-Watson | 0.345515 |

Joint test on named regressors -

Test statistic: F(1, 9) = 11.9552

with p-value = P(F(1, 9) > 11.9552) = 0.00718758

Robust test for differing group intercepts -

Null hypothesis: The groups have a common intercept Test statistic: Welch F(9, 107.0) = 409.683

with p-value = P(F(9, 107.0) > 409.683) = 1.16925e-78

**Table S12.** MPI-ESM-MR 6-Month and Annual Analysis for HCI:Combined

Model 8: Fixed-effects, using 315 observations

Included 10 cross-sectional units

Time-series length: minimum 17, maximum 44

Dependent variable: l_Overnightstay

Robust (HAC) standard errors

|  | *Coefficient* | *Std. Error* | *t-ratio* | *p-value* |  |
| --- | --- | --- | --- | --- | --- |
| const | −7.38532 | 5.55620 | −1.329 | 0.2165 |  |
| HCI_MPI_6month | 0.265394 | 0.0705967 | 3.759 | 0.0045 | *** |

| Mean dependent var | 13.50204 | S.D. dependent var | 1.734971 |
| --- | --- | --- | --- |
| Sum squared resid | 190.5809 | S.E. of regression | 0.791777 |
| LSDV R-squared | 0.798365 | Within R-squared | 0.104196 |
| Log-likelihood | −367.8225 | Akaike criterion | 757.6450 |
| Schwarz criterion | 798.9233 | Hannan-Quinn | 774.1372 |
| rho | 0.762296 | Durbin-Watson | 0.427947 |

Joint test on named regressors -

Test statistic: F(1, 9) = 14.1323

with p-value = P(F(1, 9) > 14.1323) = 0.00448992

Robust test for differing group intercepts -

Null hypothesis: The groups have a common intercept Test statistic: Welch F(9, 106.8) = 395.606

with p-value = P(F(9, 106.8) > 395.606) = 9.81914e-78

Model 6: Fixed-effects, using 315 observations

Included 10 cross-sectional units

Time-series length: minimum 17, maximum 44

Dependent variable: l_Overnightstay

Robust (HAC) standard errors

|  | *Coefficient* | *Std. Error* | *t-ratio* | *p-value* |  |
| --- | --- | --- | --- | --- | --- |
| const | −10.3203 | 5.41375 | −1.906 | 0.0890 | * |
| HCI_MPI_annual | 0.351897 | 0.0799702 | 4.400 | 0.0017 | *** |

| Mean dependent var | 13.50204 | S.D. dependent var | 1.734971 |
| --- | --- | --- | --- |
| Sum squared resid | 176.6705 | S.E. of regression | 0.762334 |
| LSDV R-squared | 0.813082 | Within R-squared | 0.169580 |
| Log-likelihood | −355.8856 | Akaike criterion | 733.7711 |
| Schwarz criterion | 775.0494 | Hannan-Quinn | 750.2634 |
| rho | 0.705207 | Durbin-Watson | 0.512085 |

Joint test on named regressors -

Test statistic: F(1, 9) = 19.3631

with p-value = P(F(1, 9) > 19.3631) = 0.00171951

Robust test for differing group intercepts -

Null hypothesis: The groups have a common intercept Test statistic: Welch F(9, 106.9) = 367.476

with p-value = P(F(9, 106.9) > 367.476) = 4.10529e-76
